# Magnetic catheter robot with dynamic force feedback for stiffness identification and abnormality detection

**DOI:** 10.1016/j.fmre.2026.03.014

**Published:** 2026-04-07

**Authors:** Haolan Shen, Zhengwei Li, Shixiong Fu, Mingguo Jiang, Mingxue Cai, Zhiqiang Chen, Hongwei Wang, Long Cheng, Tiantian Xu

**Affiliations:** a Guangdong Provincial Key Laboratory of Robotics and Intelligent System, Shenzhen Institutes of Advanced Technology, Chinese Academy of Sciences, Shenzhen 518055, China; b University of Chinese Academy of Sciences, Beijing 100049, China; c State Key Laboratory of Multimodal Artificial Intelligence Systems, Institute of Automation, Chinese Academy of Sciences, Beijing 100190, China; d The Key Laboratory of Biomedical Imaging Science and System, Shenzhen Institutes of Advanced Technology, Chinese Academy of Sciences, Shenzhen 518055, China; e Fuwai Yunnan Hospital, Chinese Academy of Medical Sciences, Affiliated Cardiovascular Hospital of Kunming Medical University, Kunming 650000, China

**Keywords:** Magnetically actuated catheter, Dynamic force feedback, Quantitative stiffness identification, Abnormality detection, Minimally invasive surgery

## Abstract

Magnetic continuum robots offer unique advantages for minimally invasive surgery owing to their flexibility, miniaturization, and remote actuation. However, the absence of reliable real-time force feedback in existing magnetically actuated catheters severely constrains safe interaction, and quantitative identification of tissue mechanical properties. To address this limitation, this work presents a magnetic catheter robot with integrated dynamic force feedback. The proposed system integrates an axial optical force sensor, a miniaturized endoscope, and annular permanent magnets within a compact distal architecture. This design achieves sub-0.01 N force resolution while remaining robust under large bending deformations and varying deflection angles. System performance is quantitatively evaluated through force-controlled planar writing, 3D curved-surface palpation with shape perception and stiffness mapping, and endoscope-guided bronchial palpation, demonstrating reliable stiffness-based abnormality detection in complex luminal environments. These results demonstrate the feasibility of force-aware magnetic catheterization for quantitative assessment of local tissue mechanics, providing a clinically relevant platform for minimally invasive diagnostics.

## Graphical abstract

**Figure:**
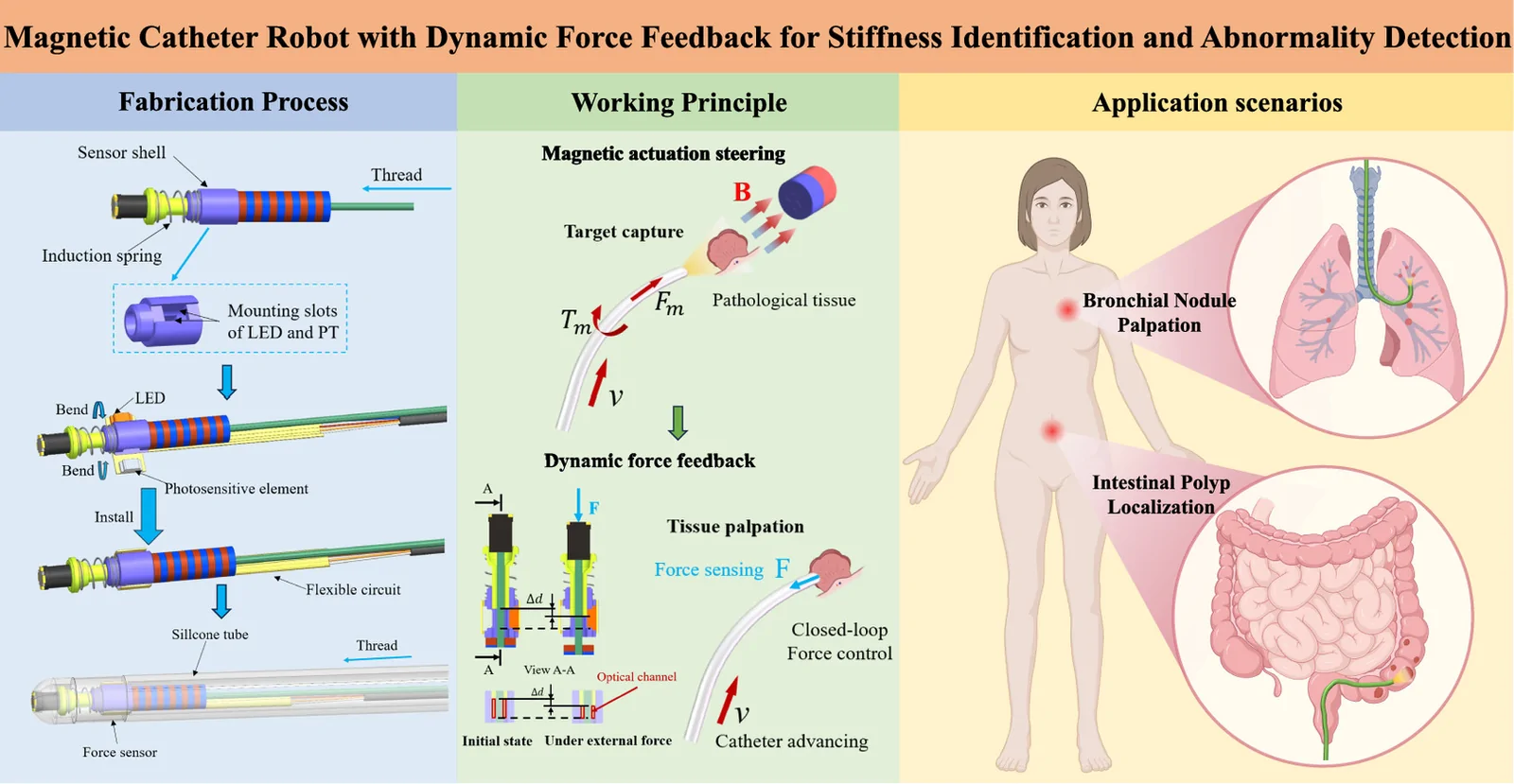

## 1 Introduction

Minimally invasive surgery (MIS) and flexible endoscopy have emerged as essential tools [1], [2], [3] for diagnosing and treating diverse luminal diseases due to their reduced tissue trauma, faster recovery, and improved clinical outcomes [4], [5], [6]. However, despite their widespread adoption, current endoscopic procedures remain predominantly vision-guided and lack quantitative feedback on tissue interaction forces. Early or subtle abnormalities, including flat, submucosal, or stiffness-altered lesions in the respiratory, gastrointestinal, and urinary tracts, often exhibit limited surface cues [7], [8]. As a result, they are difficult to detect or characterize purely from visual information. Meanwhile, the absence of real-time force and stiffness sensing forces clinicians to rely heavily on experience during endoscope manipulation. This reliance increases the risk of tissue injury and complicates safe navigation in tortuous or branching anatomies. These challenges underscore an urgent clinical demand for actively steerable, force-aware luminal robotic systems that can enhance navigation while providing quantitative information on local tissue mechanics.

Magnetic continuum robots (MCRs) have therefore distinguished themselves as a promising advancement in robotic catheter technology, enabling enhanced navigation capabilities and surgical precision through remote magnetic actuation [9], [10]. Without internal actuation components, MCRs achieve active steering under external magnetic fields [11], [12], [13], [14]. Recent research has significantly expanded the functional capabilities of MCRs. In terms of structural innovation, Nelson et al. developed fast-response, multisegmented magnetic catheters with variable-stiffness architectures, improving dexterity in confined surgical environments [15], [16], [17], [18]. Meanwhile, Liu et al. explored oppositely magnetized configurations and proposed kinematic control strategies to enhance stability in medical applications [19], [20], [21]. In the domain of teleoperated navigation, Zhao et al. demonstrated a magnetically controlled guidewire system for robotic neurointervention, enabling precise steering for cerebral aneurysm treatment [22], [23]. Xu et al. further advanced this concept by integrating magnetic steering and propulsion to improve vascular navigation efficiency in complex vascular pathways [24], [25], [26], [27]. Zhang et al. advanced magnetic continuum robots with active locomotion, programmable magnetic moment control, and real-time navigation under ultrasound or vision-based feedback, enhancing dexterity in constrained environments [28], [29], [30], [31]. Beyond navigation, MCRs are being developed for multifunctional interventions. Xie et al. introduced an MCR integrating endoscopic imaging, biopsy tools, and variable-stiffness manipulation for transluminal procedures [32], [33], while Pittiglio et al. leveraged magnetic actuation for lung endoscopy, combining targeted photothermal therapy with robotic navigation [34]. These advancemnts collectively highlight the potential of MCRs to serve as highly dexterous and versatile luminal robots.

However, most existing MCRs lack direct tip–tissue force sensing, which limits safe force regulation and stiffness-based lesion detection [23]. Existing approaches to contact force acquisition generally fall into two categories: model-based indirect estimation and sensor-integrated direct measurement. Indirect methods infer contact forces from robot deformation using kinematic or continuum-mechanics models. Wang et al. utilized finite difference methods to solve both continuum robot deformation and end-effector contact forces [35]. Dreyfus et al. used a Lagrange-multiplier simulation method to model contact interactions and obtain the corresponding forces [36]. Although these approaches avoid adding distal hardware, their accuracy is highly sensitive to modeling assumptions, material properties, and boundary conditions, making reliable force estimation difficult under the large, configuration-dependent deformations typical of magnetic continuum robots. Direct measurement methods integrate miniature force sensors at the catheter tip. Chuang et al. developed a piezoresistive micro-force sensor for tumor stiffness detection during endoscopic procedures [37]. Wang et al. created a liquid metal-based resistive force sensor for cardiac interventions [38]. Lee et al. implemented a capacitive multi-axis sensor using printed-circuit electrodes [39]. Nagatomo et al. fabricated a compact multi-axis capacitive sensor through microfluidic liquid-metal channels [40]. Gonenc et al. developed a fiber Bragg grating (FBG)-based triaxial force sensor for robotic micro-forceps in vitreoretinal surgery [41]. Li et al. achieved catheter force sensing through Fabry-PȨrot (FP) cavity length changes detected via optical fibers [42]. Although direct force sensors have demonstrated clear clinical value, most resistive and capacitive designs are difficult to integrate into magnetically actuated continuum robots with embedded endoscopic imaging. Their structural layers, electrode patterns, or fluidic channels are not compatible with the limited cross-sections required to accommodate distal magnets and an imaging channel, and their outputs are sensitive to local deformation and hysteresis during large bending. Fiber Bragg grating (FBG) sensors offer higher resolution and immunity but face system-level integration constraints in highly deformable magnetic catheters [43], [44]. Because FBGs measure strain rather than force, shape-induced strain under large bending can couple with contact-induced strain, necessitating multiple gratings or fibers and complex decoupling models, which compete for the already limited cross-sectional space. Moreover, FBG interrogation depends on bulky and expensive optical hardware, hindering integration into compact robotic systems and conflicting with the cost and disposability requirements of clinical luminal devices. In many lumen-based diagnostic tasks, clinicians primarily rely on axial contact force rather than full three-axis loads. Consequently, the added complexity of multi-axis FBG architectures does not necessarily yield proportional benefits in these applications.

To address the above challenges, this work presents a magnetic catheter robot that integrates, at its distal end, an optical force sensor, a miniaturized endoscope, and annular permanent magnets within a unified and compact architecture. The core sensing module is a single-axis optical force sensor based on an LED–phototransistor occlusion principle, enabling axial contact force to be measured with high resolution and strong robustness to catheter bending. With a minimum detectable force of 0.01 N and a hollow-core structure compatible with a 3–4 mm form factor, the sensor supports real-time force feedback without compromising endoscopic imaging or magnetic actuation. Requiring only an LED, a phototransistor, and a low-cost ADC, the module is straightforward to integrate and inexpensive to manufacture, while maintaining minimal sensitivity to catheter curvature. Leveraging this sensing capability, the robot establishes a closed-loop control framework that enables simultaneous real-time visualization and quantitative tissue-contact force feedback, thus supporting safe luminal navigation and controlled tissue–robot interaction. As illustrated in Fig. 1, this technology extends across diverse luminal interventions, including bronchial nodule palpation and other representative luminal inspection scenarios. By synergizing magnetic steering, dynamic force feedback, and quantitative stiffness analysis, this robot establishes a versatile and clinically meaningful platform for minimally invasive luminal interventions.

**Fig. 1:**
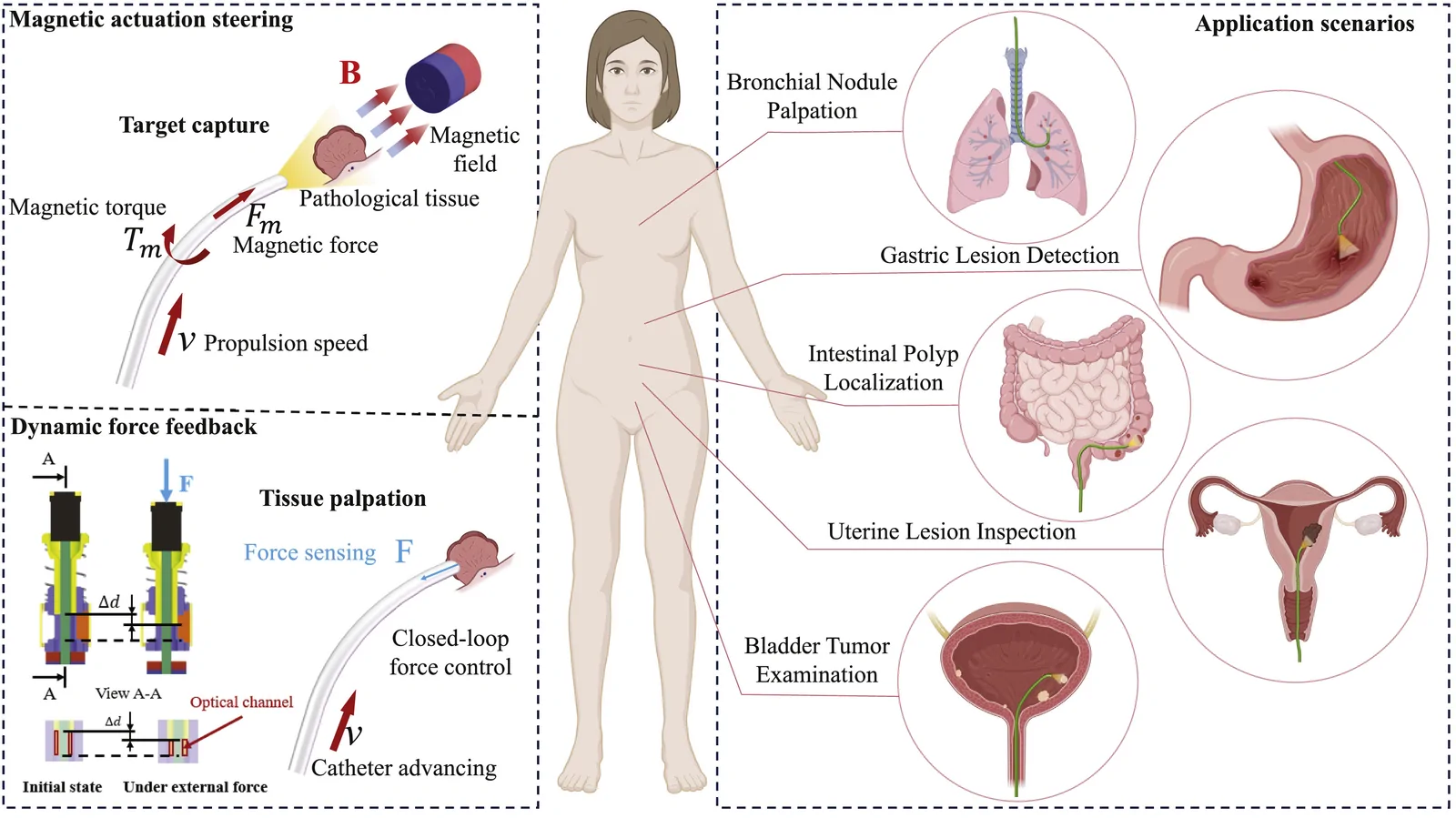
**Working principle and clinical scenarios of the magnetically actuated catheter robot.** The robot leverages magnetic force and torque for catheter steering and target capture, while the integrated closed-loop force sensing mechanism enables real-time tissue palpation with a minimum 0.01 N resolution. The proposed robot applies to diverse minimally invasive luminal interventions, including bronchial nodule palpation, gastric lesion detection, intestinal polyp localization, uterine lesion inspection, and bladder tumor examination. (All organ and human model images were created using BioRender and then processed.).

## 2 Materials and methods

### 2.1 Design and fabrication of magnetic catheter robot

Building upon the previously developed optoelectronic sensor [45], structural adaptations were necessary to enable highly sensitive force detection at the guidewire’s tip. To ensure unobstructed visualization while maintaining high-precision force sensing, the force-sensing module was integrated posterior to the camera assembly. To further address spatial constraints and signal integrity, a hollow structure was adopted, providing centralized routing for the camera signal lines. This posterior placement strategy also serves to balance force sensing performance with mechanical flexibility and size constraints at the distal end. By positioning the sensing module behind the camera and embedding part of the endoscope within the sensor housing, the rigid sensing components are not located at the extreme distal tip. The resulting axial length from the camera head to the bottom of the sensing module is approximately 7.5 mm, which remains compact relative to the catheter diameter and introduces only limited additional stiffness. Consequently, sufficient bending compliance is preserved for intraluminal navigation, while the distal tip diameter is maintained within a clinically compatible range.

The fabrication of MCR required meticulous material selection and a well-defined assembly process to ensure its functionality and structural stability. We utilized a 2 mm diameter OVM6946 miniature endoscope with an RGB camera and four distal illumination LEDs for endoscopic visualization. Polyether ether ketone (PEEK) was selected for the camera mount and sensor shell due to its excellent insulation, lightweight, machinability, and self-lubricating properties, and these components were fabricated using CNC machining. For the induction spring, we employed 304 stainless steel micro springs. These springs have an outer diameter of 2 mm, a length of 3 mm, and wire diameters of 0.08 mm, 0.12 mm, and 0.15 mm, respectively. Such specifications endow them with tunable stiffness, enabling efficient force-displacement transduction. The optoelectronic sensor incorporates 940 nm wavelength LEDs (model XL-1608IRC940, 0603 package) and phototransistors (PT, model MPT60363T, 0603 package) as the photosensitive elements, both mounted onto a flexible printed circuit (FPC). The FPC was constructed using a polyimide (PI) substrate with gold-plated copper foil conductors and a board thickness of 0.11 mm, ensuring robust electrical connectivity and mechanical flexibility. The detailed parameters of the magnetic catheter are provided in Supplementary Material Table S1.

The assembly process began with threading the camera signal line through the machined camera mount and securing it with adhesive (Fig. 2A). The camera signal line was then routed through the induction spring and sensor shell, effectively linking the two components via the induction spring. Multi-segment annular NdFeB magnets (diameter: 2 mm, height: 1 mm) was threaded through the catheter wire and inserted into a sensor shell to form the magnetic actuation unit (Fig. 2B). For optoelectronic integration, the LED and photosensitive element were soldered onto the FPC, which was carefully bent and inserted into the designated slots within the sensor shell to ensure stable fixation. To prevent displacement or misalignment, the FPC was securely adhered to the sensor shell, with output signal transmission facilitated by flexible wires soldered to the FPC (Fig. 2C). The preliminary encapsulation prototype and dimensions of the magnetic catheter robot are detailed in Fig. S2 of the Supplementary Materials. To encapsulate the catheter tip, we used a 3D printed mold to support the silicone cap (outer diameter: 3 mm), which was homogeneously cast onto the silicone cap using Ecoflex 00-30 composite material. After curing for 4 h at 25 ${}^{\circ }$C, a 0.2 mm thick elastic membrane was formed, allowing for waterproofing and dynamic deformation compatibility (Fig. 2D). The membrane and perforated silicone cap were subsequently assembled onto the endoscope, ensuring unimpeded visualization and force sensor functionality (Fig. 2E). Finally, the integrated sensor was embedded into a silicone tube (outer diameter: 4 mm) using medical-grade adhesive to ensure that the sensor shell was flush with the end of the silicone tube.

**Fig. 2:**
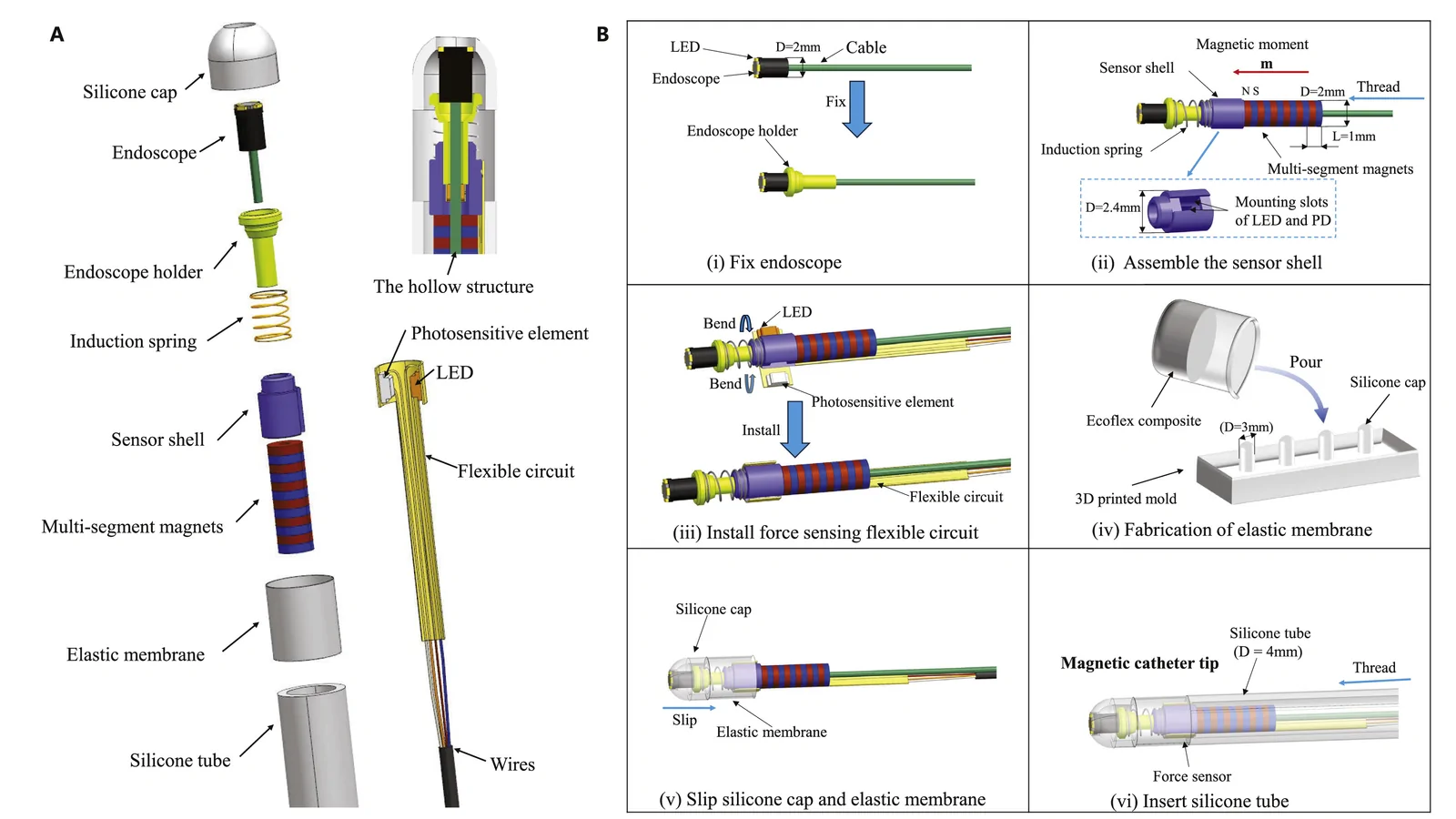
**Structure model and fabrication process of the proposed magnetic catheter robot.** (A) Exploded view of the magnetic catheter structure model. (B) Specific fabrication process for the magnetic catheter integrated with endoscope and optical force sensor. (i) The endoscope was secured as the foundation for sensor integration. (ii) The protective shell was assembled to house the sensing components, with multi-segment magnets inserted as the magnetic actuation unit. (iii) PI-based FPC with an embedded LED and phototransistor was bent and secured into sensor shell slots. (iv) A waterproof elastic membrane was fabricated using Ecoflex composite material. (v) The silicone cap and elastic membrane were fitted to the endoscope tip for protection. (vi) A flexible silicone tube was finally inserted to complete the robot.

In the initial state, as depicted in the lower left part of Fig. 1, light emitted from the LEDs passes on either side of the camera signal line and is received by the photosensitive elements. When an external force is applied to the catheter tip, its response can be analyzed from both mechanical and optoelectronic perspectives. Mechanically, the relative displacement created by the induction spring between the camera mount and the sensor shell follows Hooke’s law. Assuming that the selected spring is linear with a stiffness coefficient k and the sensor shell is fixed, the relative displacement Δd between the camera mount and the sensor shell under an applied force F can be expressed as

(1) $$\Delta d=\frac{f}{k}.$$

Optoelectronically, the compression of the induction spring induces a displacement of the camera mount, partially blocking the emitted light and reducing the received light flux. The sensor quantifies pressure-induced displacement by monitoring variations in light flux caused by the relative motion. Here, the LED is considered as a uniform surface light source. Previous research indicates that within a certain range, the output voltage of the photosensitive element has a linear relationship with the light flux [46]. Therefore, the relationship between the pressure received by the sensor and the output voltage of the photosensitive element can be formulated as

(2) $$\Delta V=\Delta d\left(L-D\right)J=\frac{J(L-D)}{k}F,$$

where J represents the linear coefficient between the sensor’s output voltage and the light flux, L is the width of the light-passing slot in the sensor shell, and D is the diameter of the camera signal line. As S,L,D and k are constants, the sensor’s output voltage exhibits a direct linear correlation with the applied force. The sensitivity and detection range of the sensor can be adjusted by changing the stiffness k of the induction spring.

### 2.2 Kinematic modeling of magnetic catheter robot

An internal permanent magnet (IPM) embedded at the catheter tip interacts with an external permanent magnet (EPM), which generates a spatially variable magnetic field $B={\left[B_{x}\;B_{y}\;B_{z}\right]}^{T}$ [47]. The resulting magnetic force $F_{m}$ and magnetic torque $\tau_{m}$ on the catheter tip can be expressed as

(3) $$F_{m}=\left(M\cdot \nabla \right)B$$

(4) $$\tau_{m}=M\times B$$

where $M={\left[M_{x}\;\;M_{y}\;\;M_{z}\right]}^{T}$ denotes the IPMs magnetic dipole moment. By applying Maxwells equations and standard simplifications, the Eqs. (3) and (4) could be rewritten in matrix form as

(5) $$\begin{array}{cc} F_{m} & ={\left[\begin{array}{ccc} \frac{\partial B}{\partial x} & \frac{\partial B}{\partial y} & \frac{\partial B}{\partial z} \end{array}\right]}^{T}M\\ & =\left[\begin{array}{ccc} \frac{\partial B_{x}}{\partial x} & \frac{\partial B_{x}}{\partial y} & \frac{\partial B_{x}}{\partial z}\\ \frac{\partial B_{x}}{\partial y} & \frac{\partial B_{y}}{\partial y} & \frac{\partial B_{y}}{\partial z}\\ \frac{\partial B_{x}}{\partial z} & \frac{\partial B_{y}}{\partial z} & -\;\left(\frac{\partial B_{x}}{\partial x}+\frac{\partial B_{y}}{\partial y}\right) \end{array}\right]\left[\begin{array}{c} M_{x}\\ M_{y}\\ M_{z} \end{array}\right] \end{array}$$

(6) $$\tau_{m}=\left[\begin{array}{ccc} 0 & B_{z} & -B_{y}\\ -B_{z} & 0 & B_{x}\\ B_{y} & -B_{x} & 0 \end{array}\right]\left[\begin{array}{c} M_{x}\\ M_{y}\\ M_{z} \end{array}\right]$$

The magnetic field at the catheter tip, generated by the EPM, can be approximated by a magnetic dipole model under a non-uniform field assumption [48]:

(7) $$B\left(r,M_{E}\right)=\frac{\mu_{0}}{4\pi r^{5}}\left(3rr^{T}-r^{2}I_{3}\right)M_{E}$$

where $r=P_{I}-P_{E}$ is the vector from the EPM to the IPM, r=∥r∥, $\mu_{0}=4\pi \times 10^{-7}\;T\cdot m\cdot A^{-1}$ is the vacuum permeability, $I_{3}$ is the 3×3 identity matrix, and $M_{E}$ is the magnetic dipole moment of the EPM.

The pose of the EPM is commanded directly in Cartesian space. The UR10 robotic arm internally computes the inverse kinematics required to achieve the specified end-effector pose. Therefore, the magnetic field $B(P_{I})$ and its gradient $\nabla B(P_{I})$ are fully determined by the commanded magnet trajectory, without requiring analytical inverse-kinematics derivations within our modeling framework.

In the steering workspace, the external permanent magnet generates a magnetic field with minimal gradients, so the magnetic force term (M·∇)B acts primarily along the catheter axis and does not contribute to bending. Accordingly, only the torque term M×B enters the subsequent curvature model [16], [47].

To model the catheters mechanical response, we employ the Euler-Bernoulli beam theory, which assumes that plane sections remain plane and perpendicular to the neutral axis during deformation [16]. Under Euler–Bernoulli beam assumptions, axial loading does not induce curvature, whereas the moment generated by the magnetic torque is solely responsible for bending of the distal segment. The curvature κ of the catheter is determined by the bending moment generated by the magnetic torque acting in the bending plane, and is given by

(8) $$\kappa =\frac{\tau_{m}}{EI}$$

where E is the Youngs modulus of the catheter material, I is the second moment of area of the catheter cross-section and $\tau_{m}=\left|\tau_{m}\right|$ denotes the magnitude of the applied magnetic torque computed from Eq. (4).

To relate the magnetic torque–induced curvature to the resulting catheter deflection, the distal flexible segment is modeled using the standard piecewise-constant-curvature (PCC) assumption, which is widely adopted in continuum-robot kinematics [49], [50]. Under this assumption, each bending segment is approximated as an arc of constant curvature κ. The tip orientation $\theta_{\mathrm{tip}}$ relative to the proximal segment is therefore given by

(9) $$\theta_{\text{tip}}=\kappa L$$

where L is the effective bending length of the catheter. The corresponding tip position in the bending plane is

(10) $$x=\frac{1}{\kappa }\left(1-\cos\left(\kappa L\right)\right)$$

(11) $$y=\frac{1}{\kappa }\sin\left(\kappa L\right)$$

which provide the spatial deflection associated with curvature κ.

Under magnetic actuation, the IPM experiences a torque that tends to align its magnetic moment with the applied magnetic field. Only the torque component acting in the bending plane contributes to catheter deflection. Let θ denote the magnetic-field orientation in this plane, and $\theta_{\mathrm{tip}}$ the catheter-tip orientation defined in Eq. (9). The magnitude of the effective bending torque can therefore be written as

(12) $$\tau_{m}=BM\sin\left(\theta -\theta_{\mathrm{tip}}\right)$$

where B=∥B∥ and M=∥M∥ are the magnitudes of the magnetic moment and magnetic field, respectively. This expression follows directly from the standard dipole torque relation Eq. (4) and projects the torque onto the bending plane.

Substituting Eq. (12) into the curvature expression Eq. (8) and then into the PCC orientation relation Eq. (9) yields the implicit equilibrium equation governing the steady-state bending configuration:

(13) $$\theta_{\mathrm{tip}}=\frac{LBM}{EI}\sin\left(\theta -\theta_{\mathrm{tip}}\right)$$

This equation describes the balance between the magnetic torque that attempts to align the IPM with the external field and the elastic restoring moment of the catheter. It provides a complete mapping from the applied magnetic-field orientation and magnitude to the resulting tip orientation predicted by the PCC model.

### 2.3 Calibration experiment of optical force sensor

We tested the sensor’s performance using a custom-built test platform with an ATI six-axis force sensor as the reference, as shown in Fig. 3A. All mechanical calibration experiments were performed in a controlled laboratory environment at $25\pm 1{\;}^{\circ }\text{C}$ and (50±5)% relative humidity. The sensor’s sensitivity is represented by the gauge factor (computed by determining the slope of the linear fit) $S=(\Delta U/U_{\text{min}})/\Delta F$. Here, U represents the output voltage of the sensor, $U_{\text{min}}$ is the stabilizing voltage for the minimum output of the sensor, and F denotes the applied force. The linearity ($R^{2}$) value is computed from the coefficient of determination of the linear fit within each operating force range. It should be emphasized that the wire diameter of the induction spring is treated as a tunable design parameter rather than a fixed optimal choice. Springs with different diameters were intentionally selected to characterize the sensor performance across distinct force ranges and experimental requirements. Initially, we tested the sensor’s sensitivity using a induction spring with a wire diameter $W_{d}=0.08$ mm (The variation of the relative voltage output by the sensor with the force is represented by orange dots and the black solid line represents the linear fitting curve, as shown in Fig. 3B). The experimental test sensitivities of the sensor under force are 30,770 N${}^{-1}$ (with an $R^{2}$ of 0.9724 in the operating range of 0–0.025 N). The experimental results showed that the sensor has extremely high sensitivity and good linearity, validating our theoretical analysis. Notably, these calibration results are obtained with the sensing module positioned posterior to the camera, demonstrating that this configuration preserves the basic force transduction performance and sensitivity of the sensor.

**Fig. 3:**
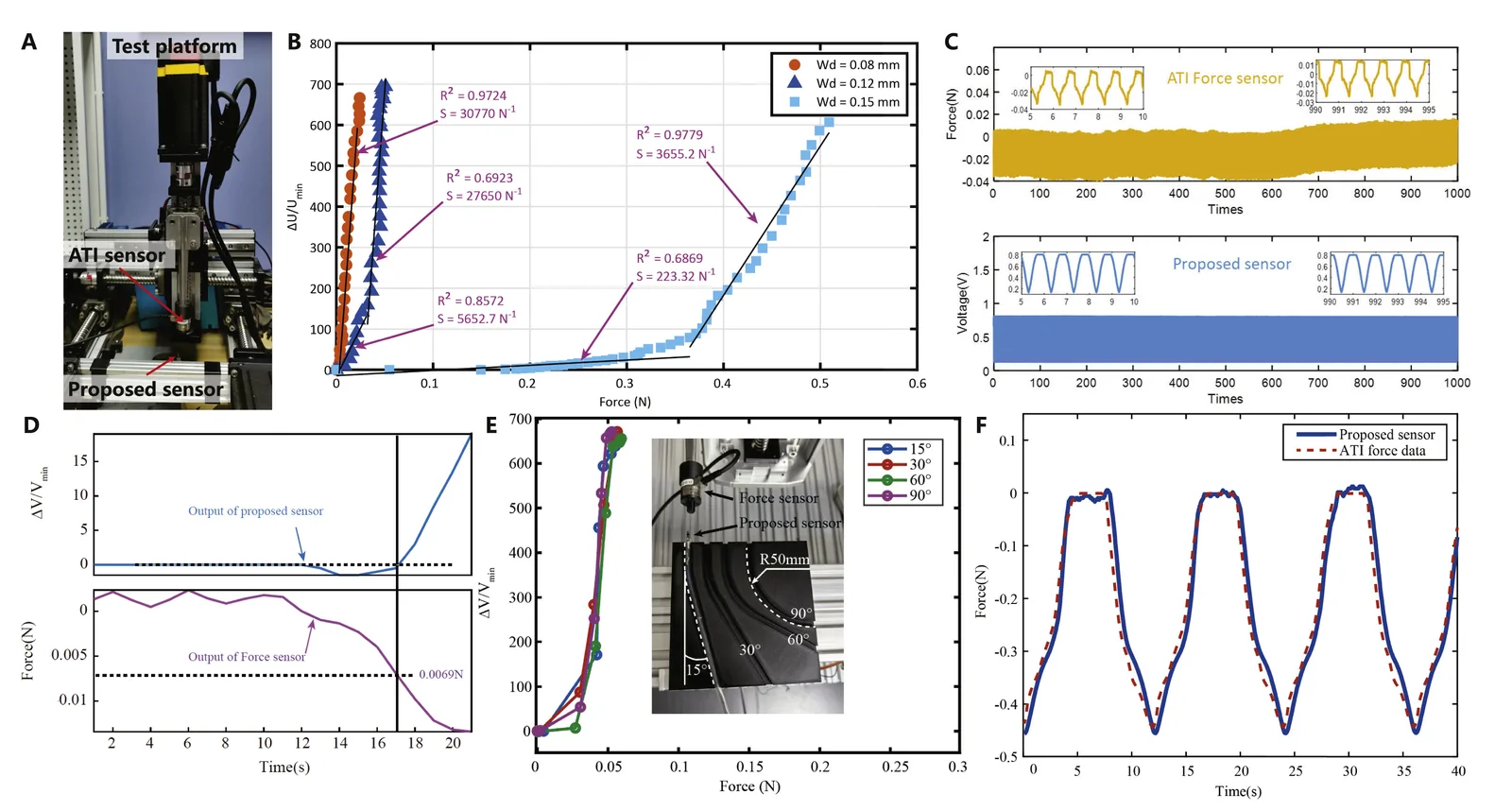
**Mechanical calibration and performance evaluation of the optical force sensor.** (A) Experimental setup for mechanical calibration. (B) Sensitivity and measurement range evaluation of springs with different wire diameters. (C) Stability comparison between the proposed force sensor and an ATI six-axis force sensor. (D) Force resolution evaluation of the proposed sensor using an ATI reference force sensor. (E) Force calibration of the proposed sensor under different catheter bending angles. (F) Comparison of axial force measurements between the proposed sensor and an ATI force sensor.

In addition, we have also tested the sensitivity and measurement range of the sensor when different springs with varying wire diameters are selected for the sensor. For $W_{d}=0.12$ mm, the experimental test sensitivities (represented by blue triangles in Fig. 3B) are 5652.7 N${}^{-1}$ (with an $R^{2}$ of 0.8572 in the operating range of 0–0.025 N) and 27,650 N${}^{-1}$ (with an $R^{2}$ of 0.6923 in the operating range of 0.025–0.05 N). For $W_{d}=0.15$ mm, the experimental test sensitivities (represented by cyan squares in Fig. 3B) are 223.32 N${}^{-1}$ (with an $R^{2}$ of 0.6869 in the operating range of 0–0.36 N) and 3655.2 N${}^{-1}$ (with an $R^{2}$ of 0.9779 in the operating range of 0.36–0.5 N). It can be seen from the experimental results that when $W_{d}=0.12$ mm and $W_{d}=0.15$ mm, the sensitivity curve of the sensor shows a piece-wise linear pattern. This is because, in the initial stage of the sensor, the induction spring is pre-compressed, and the camera signal line is stretched. At this time, the camera signal line can be regarded as an equivalent spring. When an external force acts on the sensor, the induction spring is further compressed, and the equivalent spring is released, which corresponds to the first stage of the sensor. When the external force continues to increase, the endoscope signal line returns to a free state, and the equivalent spring disappears, which corresponds to the second stage of the sensor, as analyzed in Fig. S1 of the Supplementary Materials. In future work, we will eliminate this nonlinearity by adding non-stretchable fibers to the surface of the camera signal line.

Next, we tested the stability performance of the sensor and selected $W_{d}=0.08$ mm as the induction spring, as shown in Fig. 3 C. We compared the stability performance of the sensor proposed in this paper and a commercial sensor (ATI nano 17) after cyclic pressing/releasing. The experimental results show that after 1000 consecutive pressing-releasing operations (within the range of 0–0.02 N), for the proposed sensor, the upper bound of the output signal drifts by 4.34%, and the lower bound of the output signal drifts by 4.41%. While for the commercial sensor, the upper bound of the output signal drifts by 43.4%, and the lower bound of the output signal drifts by 44.1%. This result indicates that the sensor proposed in this paper has good stability. Given the bonded, axially aligned integration of the endoscope and sensing module, the mechanical force transmission path from the distal tip to the sensing unit is minimized. Combined with the rapid electronic response of the LED–phototransistor pair and sampling rate (90–100 Hz), this configuration ensures that posterior placement introduces no perceptible delay in force signal transmission during real-time palpation. For the induction springs with $W_{d}=0.12$ mm and $W_{d}=0.15$ mm, the sensor structure is the same, and the measurement will not be repeated here.

To characterize the minimum detectable force and effective resolution of the proposed sensor, we conducted an additional calibration experiment using an ATI force sensor as a reference, as shown in Fig. 3D. A slight non-monotonic response is observed at very low force levels, whereas a stable and monotonic voltage response is obtained once the applied force exceeds approximately 0.0069 N. These results indicate that the effective force resolution of the proposed sensor is better than 0.01 N, which is sufficient for resolving subtle force variations encountered during soft tissue palpation. Given that the sensing module is positioned posterior to the camera, we further evaluated whether catheter bending influences axial force transmission. Force calibration curves were obtained at bending angles of 15${}^{\circ }$, 30${}^{\circ }$, 60${}^{\circ }$, and 90${}^{\circ }$ using 3D-printed arc-shaped guiding tracks that constrain the catheter to predefined curvatures while maintaining axial loading, as shown in Fig. 3E. Across all tested configurations, the force–output relationships remain monotonic and exhibit comparable sensitivity. Notably, even at a bending angle of 90${}^{\circ }$, the calibration curve closely aligns with those obtained at smaller angles, indicating that axial load transmission to the posterior sensing module is well preserved under significant curvature. Finally, to validate force measurement accuracy under dynamic axial indentation, the force measurements obtained by the proposed sensor were compared with those from the ATI force sensor, as shown in Fig. 3F. The two measurements exhibit close agreement in both waveform shape and peak magnitude, confirming that the posteriorly placed sensing module provides accurate and reliable axial force measurements during cyclic palpation. This ground-truth validation establishes the accuracy of the force signals used in the subsequent real-time force-control experiments, as shown in Fig. 4D.

**Fig. 4:**
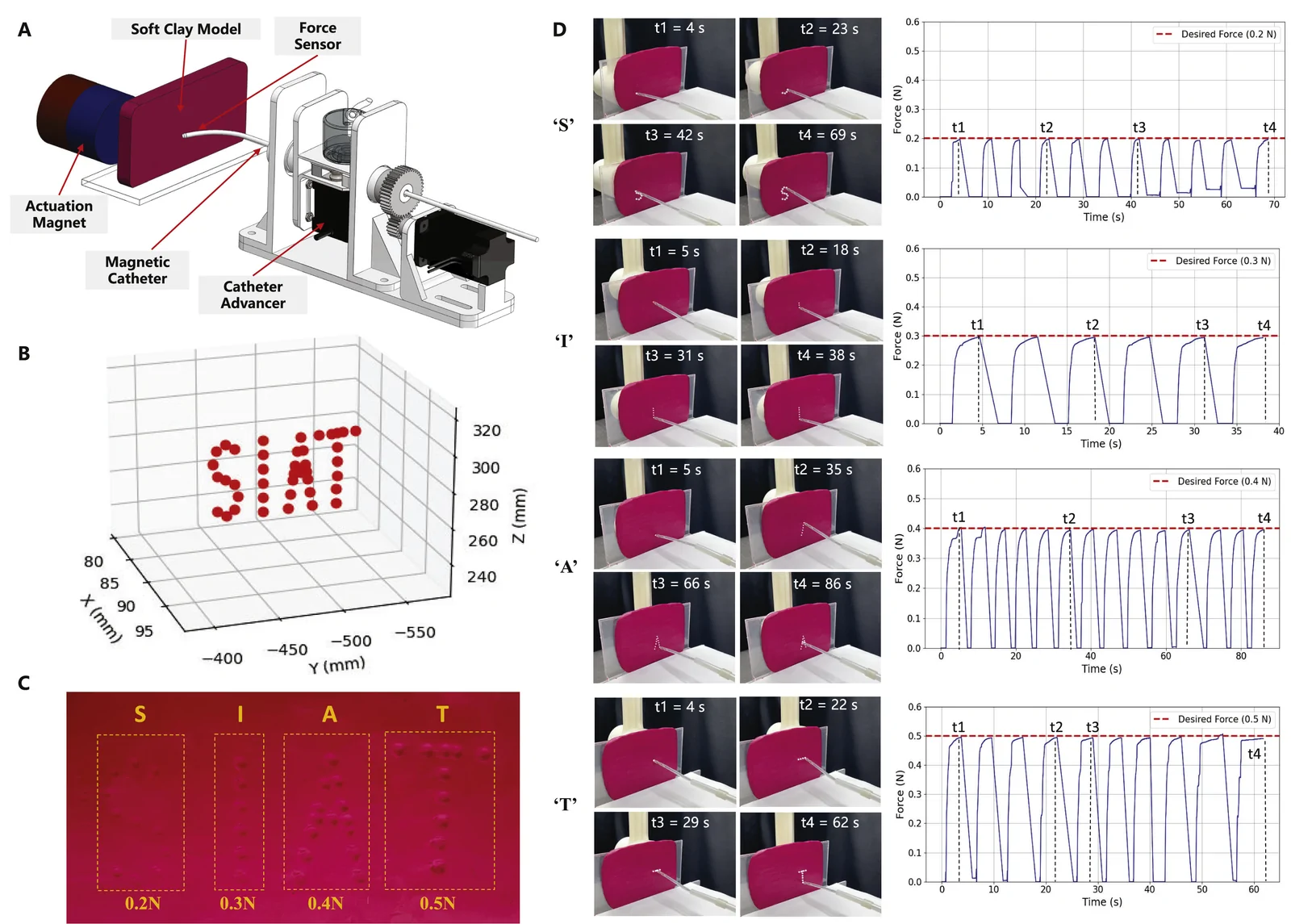
**Precision force control-based writing and indentation depth characterization on soft clay surface.** (A) Schematic illustration of magnetic catheter writing under different desired forces. (B) Desired path of the magnet pose at the end of the robotic arm. (C) Indentation depth variations in soft clay under different constant forces during the point-touch writing process. (D) Closed-loop force regulation for writing letters ‘S’, ‘I’, ‘A’, and ‘T’ on a soft clay surface, with corresponding force profiles for each letter.

To ensure that the sensor performance meets the requirements of our intended soft-tissue interaction tasks, we aligned the selection of induction springs with the expected force ranges in subsequent experiments. The spring with $W_{d}=0.15$ mm provides a calibrated and monotonic response across 0–0.36 N (and a secondary linear segment up to 0.5 N), which fully covers the operational force ranges observed in tissue palpation, stiffness probing, and catheter–tissue interactions. This mapping between spring selection and physiological force levels ensures that the sensor operates within its characterized sensitivity regions during all experiments. Furthermore, the chosen sampling rate of 90–100 Hz provides a balance between real-time responsiveness and system stability, and is sufficient to capture force variations encountered during palpation, navigation, and controlled indentation in minimally invasive procedures.

## 3 Results and discussion

### 3.1 Experiment setup

The magnetic catheter actuation platform is composed of a flexible magnet-mounted endoscope with an optical force sensor, a magnetic actuation system, and a catheter advancer. The control block diagram is shown in Fig. S3 of the Supplementary Materials. The magnetic actuation system features an axially magnetized N52-grade cylindrical NdFeB permanent magnet (60 mm in diameter and 60 mm in thickness) mounted on the UR10 robotic manipulator. The catheter advancer incorporates a linear propulsion motor and a rotary motor, enabling 2-DOF motion and accommodating the outer diameter of the catheter ranging from 2 to 5 mm. The details of the magnetic actuation system and the catheter advancer are illustrated in Figs. S4 and S5 of the supplementary materials. A computer-based interface transmits user commands to the robot while acquiring real-time force and motion data via a USB serial port. Additionally, a PyQt5-based visualization interface intuitively presents the robot’s state and force parameters, facilitating real-time monitoring and control. Experimental evaluation confirmed the control frequency of approximately 90–100 Hz. The experimental workflow consists of three stages. First, 2D planar writing under precise force control is conducted to evaluate accuracy and stability on a flexible tissue-mimicking surface. Next, shape perception and lump detection on a 3D curved surface are performed using constant force control to assess lesion localization and abnormality detection in complex environments. Finally, bronchial steering and palpation experiments are carried out to detect abnormalities such as nodules or tumorigenic regions, validating the robots capability for stiffness identification in respiratory diagnostics.

### 3.2 Planar writing on soft clay surface

In clinical practice, many minimally invasive procedures, such as endometrial biopsy and tissue sampling, rely heavily on the operator’s tactile perception. However, the inherent lack of quantifiable haptic feedback, coupled with significant inter-patient variability in tissue stiffness, poses challenges to the repeatability and precision of these procedures. To systematically evaluate the force control capability of the magnetically actuated catheter robot during tissue interaction, a 2D planar writing experiment was conducted using a soft clay tissue model.

As shown in Fig. 4A, a soft clay surface was used to simulate human soft tissue, with viscoelastic properties enabling reliable plastic deformation under external force. The magnetic catheter tip was magnetically steered by an external mobile magnet, while the optical integrated force sensor continuously measured the contact force and transmitted real-time feedback to the upper computer. A proportional-derivative (PD) control algorithm implemented on the upper computer dynamically adjusts the forward and backward movement of the catheter advancer, ensuring stable and precise force application over a range of different deflection angles. As depicted in Fig. 4B, a predefined trajectory was designed for the UR10 robotic arm to control the steering of the magnetic catheter tip for the writing task. Under controlled force conditions of 0.2 N, 0.3 N, 0.4 N, and 0.5 N, respectively, the catheter successfully wrote the letters ‘S’ ‘I’ ‘A’ and ‘T’ on a clay surface. The final traces left on the clay surface are shown in Fig. 4C, where a progressive increase in the indentation depth and clarity of the writing marks can be observed as the applied force increased. Although the commanded force levels from 0.2 to 0.5 N span both calibrated linear segments for the $W_{d}=0.15$ mm configuration, the sensors monotonic and segment-wise calibrated response allows these discrete target forces to be reliably distinguished. This is sufficient for maintaining stable force control during planar writing tasks.

During the experiment, the robot regulated contact force in real time using closed-loop feedback. The contact point sequences at target force for each letter must have a dwell time of 1 s to ensure consistent indentation formation. ‘S’ ‘I’ ‘A’ and ‘T’ were written under respective force conditions, resulting in 11, 6, 14, and 10 contact points, respectively. Fig. 4D further highlights representative points during the writing process and illustrates the spatial distribution of contact points for different letter paths. Quantitative point-to-curve error metrics, including mean error and RMSE for each letter, are summarized in Table S2, with corresponding overlay visualizations provided in Fig. S6 of the Supplementary Material. The complete experimental procedure is provided in Supplementary Movie S1. This experiment quantitatively verified that the magnetic catheter robot with integrated optical force sensing achieves real-time force control accuracy, providing a technological foundation for enhancing reliability in tissue sampling.

### 3.3 Shape perception and hard lump detection on 3D curved surface

Subcutaneous lumps often exhibit normal surface morphology, making them difficult to distinguish visually. However, pathological tissues typically exhibit greater stiffness than surrounding healthy tissues. To further evaluate the haptic perception capability of the magnetically actuated catheter on complex 3D surfaces, a palpation experiment was conducted using a biomimetic soft tissue phantom embedded with hard lumps. The experiment setup is shown in Fig. 5A. A soft arched phantom made of Ecoflex material was vertically mounted on a transparent support panel, and an automated magnetic catheter with real-time force feedback was used to perform controlled palpation. The detailed structure of the phantom is illustrated in Fig. 5B, where the maximum convex height is 16 mm and the total length is 120 mm. Prior to the experiment, two predefined palpation paths, Path 1 and Path 2, were established based on the magnet pose of the UR10 robotic arm, each consisting of 20 evenly spaced discrete contact points along an 80 mm path. To simulate the presence of subcutaneous abnormalities, rigid red spheres with diameters of 10 mm and 12 mm were embedded 2 mm beneath the surface at three-quarters of the arc in Path 1 and one-quarter of the arc in Path 2, respectively.

**Fig. 5:**
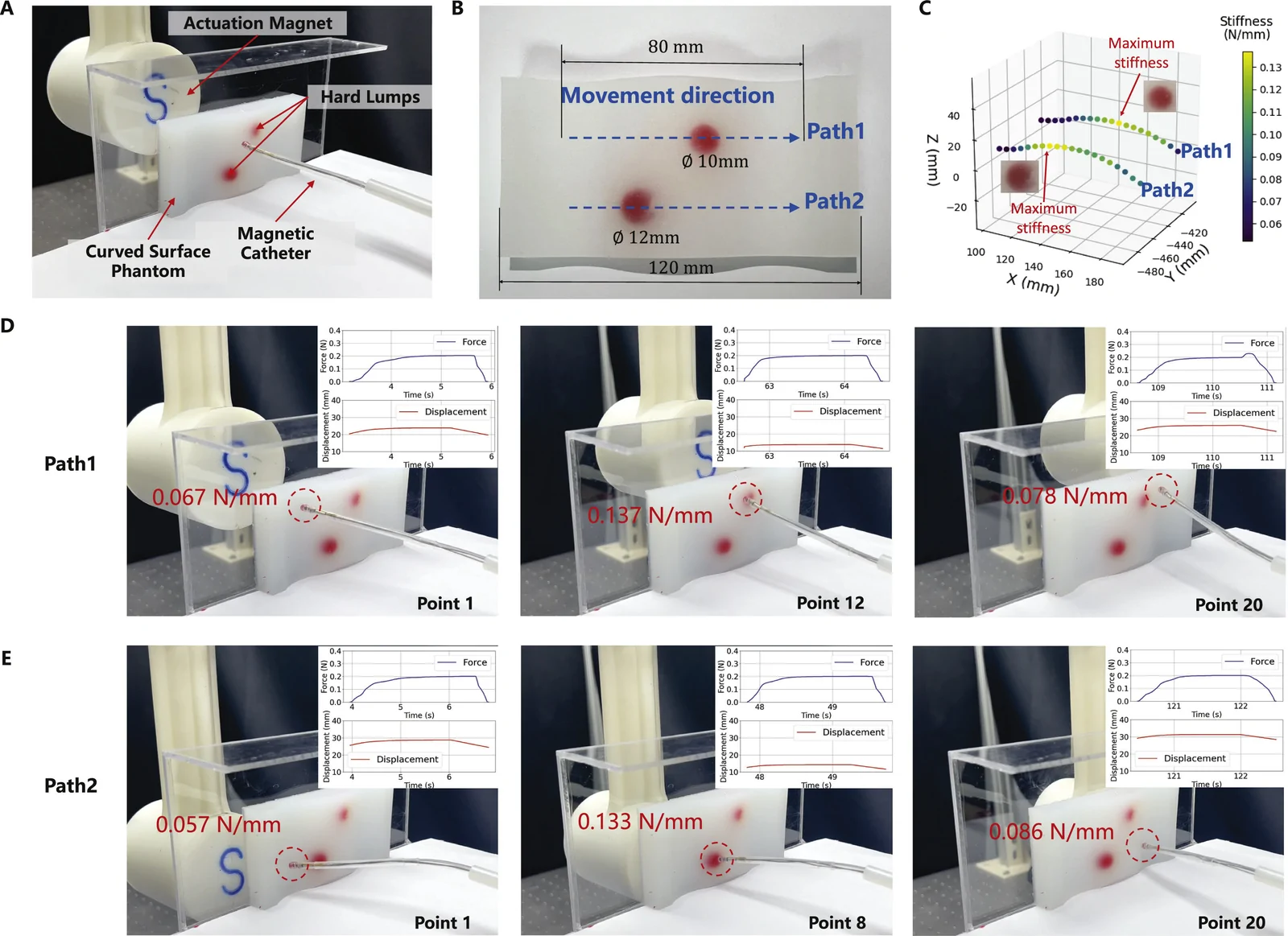
**Magnetic catheter robot with force feedback for shape perception and hard lump detection on 3D curved surface.** (A) Experiment setup of the magnetically actuated catheter with force sensing. (B) 3D curved surface phantom embedded with hard silicone spheres of different diameters. (C) Contour and stiffness distribution map derived from force-displacement data. (D, E) Force feedback and displacement variations at key points along two palpation paths under 0.2 N constant force control.

The target probing force of approximately 0.2 N lies entirely within the primary calibrated linear range from 0 to 0.36 N for the $W_{d}=0.15$ mm spring. This ensures accurate and repeatable stiffness discrimination between soft background material and embedded hard lumps. During the experiment, the optical force sensor-integrated magnetic catheter maintained a constant contact force of 0.2 N using a closed-loop force control algorithm while the absolute displacement of the catheter advancement was continuously measured by an encoder. Local stiffness was calculated as the ratio of applied force to indentation depth, enabling differentiation between soft tissue and hard lumps. Specifically, the catheter tip was controlled to gradually increase force from 0 N to 0.2 N, and indentation depth was determined by measuring the difference between the initial displacement at 0 N and the absolute displacement at 0.2 N. As shown in Fig. 5C, the results successfully detected both hard lumps, with deeper colors indicating lower stiffness values and brighter yellow colors representing higher stiffness values. The maximum stiffness was observed at contact point 12 in Path 1 and contact point 8 in Path 2. Additionally, since the rigid sphere in Path 2 was larger in diameter than that in Path 1, the stiffened region in Path 2 was noticeably larger, indicating that larger lumps induce more pronounced stiffness variations. By recording the absolute displacement values from the encoder at each contact point and computing their relative difference with respect to the highest convex point, the entire palpation paths and surface contours of the phantom were successfully reconstructed. Quantitative evaluation of the reconstructed surface profiles, including RMSE, MAE, and shape perception accuracy with respect to the CAD reference geometry, is provided in Fig. S7 of the Supplementary Material.

Fig. 5 D and E further illustrate the key stages of the palpation process for palpation path 1 and palpation path 2, including the initial position, lump contact point, and final position. Under a constant contact force of 0.2 N, the stiffness values measured in Path 1 (Fig. 5D) at the starting point, lump contact point, and final point were 0.067 N/mm, 0.137 N/mm, and 0.078 N/mm, respectively, with maximum and minimum indentation depths of 3.0 mm and 1.5 mm. Similarly, in Path 2 (Fig. 5E), the stiffness values were 0.057 N/mm, 0.133 N/mm, and 0.086 N/mm, with a maximum indentation depth of 3.5 mm and a minimum of 1.5 mm. These findings confirm that stiffer or thicker tissue regions exhibit shallower indentation depths than softer regions. Meanwhile, the upper right corner of the experimental figure shows the real-time force and displacement variations during the palpation. The complete experimental procedure is provided in Supplementary Movie S2. These results demonstrate the ability of the magnetically actuated catheter with integrated optical force sensor to effectively localize abnormal tissue regions and reconstruct tissue contours, offering valuable insights for early intraluminal lesion diagnosis.

To validate the integrated performance of the magnetically actuated catheter robot in endoscopic imaging, force sensing, and magnetic steering, we designed an experiment to evaluate its applicability in bronchial palpation, as illustrated in Fig. 6A. The detailed view highlights the key components of the magnetic catheter robot, including endoscopic imaging and an optical force sensor, which enable real-time visual and haptic integration. Prior to the experiment, a high-fidelity bronchial phantom was custom-fabricated (Fig. 6B). The phantoms outer layer was composed of soft elastomer material to replicate the tactile properties of human bronchial tissue, while internally embedded rigid nodules created a distinct mechanical contrast with the surrounding soft tissue, simulating pathological bronchial conditions. Three predefined palpation paths were designed to evaluate tissue stiffness: Path 1 targeted normal tissue at the central bronchial bifurcation, while Path 2 and Path 3 targeted rigid nodules in the left and right bronchial branches, mimicking pathological lesions. During the experiment, the operator controlled the catheter advancement speed using a teleoperation joystick. The catheter advancer ensured stable navigation to the target region, while twelve independent control buttons adjusted the magnet pose, enabling precise steering along bifurcations. Additionally, a dedicated button was programmed to automatically advance the catheter tip by 2 mm upon contact with the target tissue, leveraging absolute displacement feedback from the encoder. The catheter then retracted, and the force peak was recorded to quantify tissue stiffness.

**Fig. 6:**
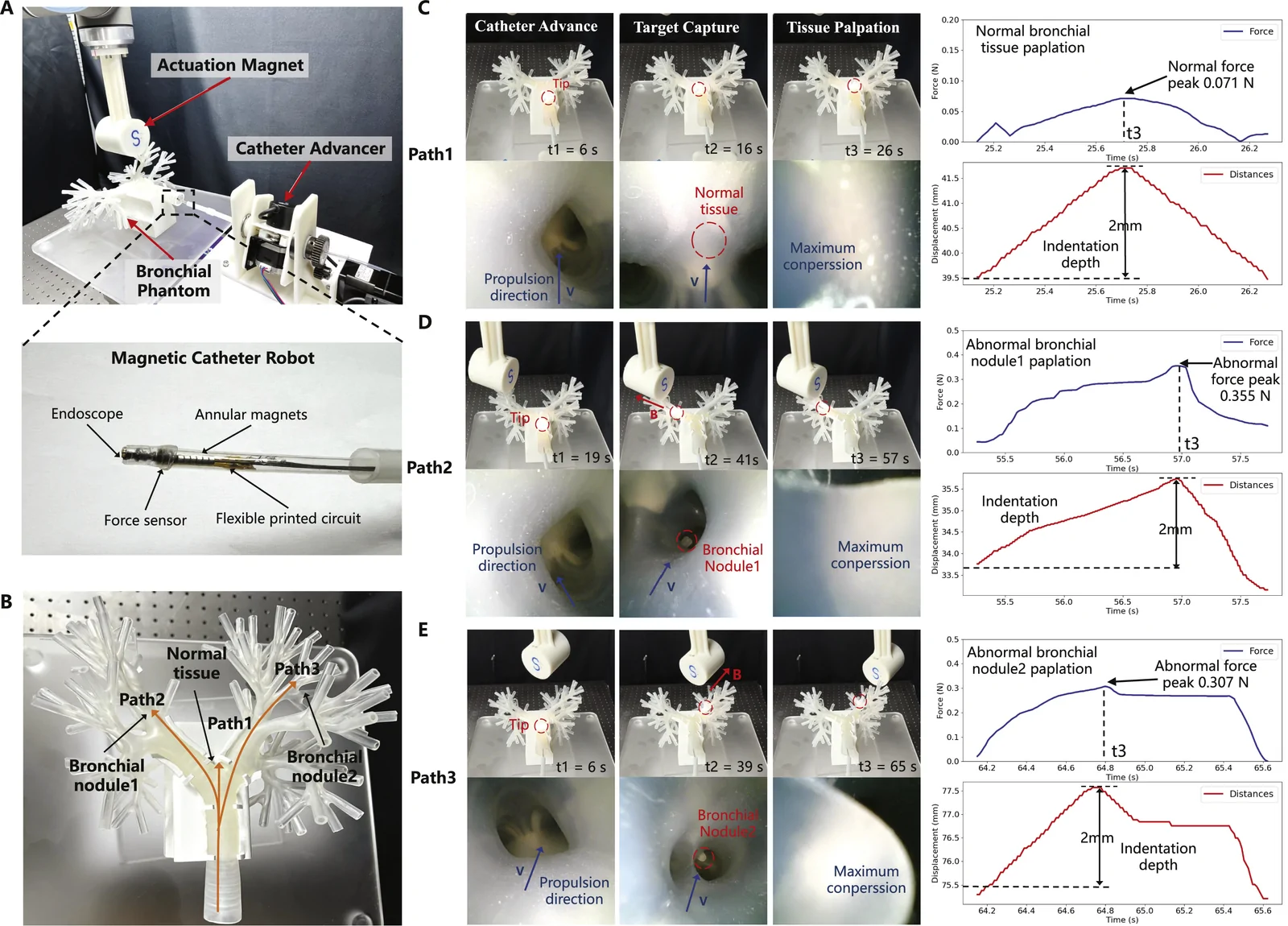
**Multimodal feedback-guided palpation in a bronchial phantom for nodule detection.** (A) Experiment setup including the 6-DOF actuation magnet, catheter advancer, the magnetically actuated catheter with integrated endoscopic imaging and force sensing, and the bronchial phantom. (B) Top view of predefined palpation paths and the target tissues corresponding to each path. (C–E) Multimodal feedback during palpation includes the external operation view and endoscope imaging of the catheter under different paths and the force-displacement profiles for quantitative 2 mm displacement palpation.

To further examine the generalizability of the proposed force-sensing magnetic catheter beyond phantoms, we additionally conducted a preliminary palpation experiment on ex vivo porcine kidney tissue as a representative biological substrate. In this experiment, hard silicone lumps of different sizes were embedded into the kidney tissue to emulate localized stiffness variations commonly associated with pathological lesions. The results indicate that the proposed system is able to distinguish regions with embedded lumps from surrounding normal tissue based on force response differences. Detailed experimental setup, sample preparation, and quantitative results are provided in Fig. S9 of the Supplementary Material.

### 3.4 Magnetic steering and nodule palpation in a bronchial phantom

Fig. 6 C–E illustrate the bronchial palpation procedure with three critical phases: catheter advance, target capture, and tissue palpation. With the assistance of endoscopic imaging, the operator can visually observe the bronchial structure in real time and dynamically differentiate morphological features between normal tissue and abnormal nodules. The force feedback profile displayed on the right quantifies the full-cycle mechanical response from initial tissue contact to completion of standardized 2 mm palpation displacement and retraction. All bronchial palpation experiments were performed under quasi-static conditions with a controlled axial indentation of 2 mm, directly measured by the encoder. Endoscopic visualization ensured axial contact and prevented lateral slipping, while magnetic actuation was completed prior to palpation and remained stationary during force measurement. These conditions ensure that the measured force–displacement profiles predominantly reflect axial indentation responses, providing stable and repeatable stiffness estimates. All measured forces, including the maximum peak of 0.355 N, fall within the calibrated linear range of the selected spring configuration, ensuring that the observed stiffness contrasts reliably distinguish rigid nodules from surrounding soft tissue. Accordingly, the localized force peaks observed in Fig. 6C–E correspond directly to regions of elevated stiffness, which are the mechanical cues used for lesion detection and assessment in bronchoscopy.

Upon catheter tip-tissue contact, the integrated optical force sensor dynamically measured the contact force, providing a quantitative stiffness assessment of the examined tissue. For Path 1 (Fig. 6C), targeting normal tissue, the optical force sensor recorded a peak force of 0.071 N under 2 mm displacement, corresponding to a stiffness of 0.0355 N/mm, with a total palpation duration of 26 s. In contrast, Path 2 (Fig. 6D, left nodule) and Path 3 (Fig. 6E, right nodule) required 57 s and 65 s, respectively, with significantly higher peak forces of 0.355 N and 0.307 N, resulting in stiffness values of 0.1775 N/mm and 0.1535 N/mm. The complete experimental procedure is provided in Supplementary Movie S3. The experiment results confirm that the magnetic catheter robot, integrating endoscopic imaging and force sensing, quantitatively differentiates normal and pathological tissues based on real-time force feedback, demonstrating its potential for minimally invasive pulmonary interventions.

## 4 Conclusion

This study introduces a novel magnetic catheter robot with integrated dynamic force feedback, improving the inherent limitation of force sensing for MCRs. By innovatively combining optical force sensing, endoscopic imaging, and magnetic steering, the magnetic catheter achieves abnormality detection, quantitative stiffness characterization, and enhanced magnetic navigation. The hollow structure design resolves spatial conflicts between sensing and imaging modalities, while tunable micro springs enhance adaptability to diverse clinical loads. Experimental validation demonstrated the high resolution of 0.01 N and confirms the robots ability for quantitative tissue mechanics assessment in complex environments. Planar writing experiments confirmed precise force regulation, 3D curved surface shape perception and lump detection successfully differentiated hard lumps from soft tissue, while bronchial nodule palpation validated its magnetic steering capability and ability to evaluate pathological stiffness variations. These results demonstrate the feasibility of integrating magnetic maneuverability with force-based tissue interaction sensing for minimally invasive robotic interventions. Future work will focus on further sensor miniaturization, enhanced automated tissue characterization, and potential extension of the optoelectronic architecture toward multi-axis force sensing when clinically required, thereby broadening the applicability of the system to diverse minimally invasive procedures.

## CRediT authorship contribution statement

**Haolan Shen:** Conceptualization, Data curation, Formal analysis, Investigation, Methodology, Project administration, Software, Validation, Visualization, Writing – original draft, Writing – review & editing. **Zhengwei Li:** Conceptualization, Data curation, Formal analysis, Validation, Visualization, Writing – original draft. **Shixiong Fu:** Conceptualization, Formal analysis, Methodology, Writing – review & editing. **Mingguo Jiang:** Conceptualization, Investigation, Visualization. **Mingxue Cai:** Conceptualization, Formal analysis, Resources, Supervision. **Zhiqiang Chen:** Conceptualization, Formal analysis, Software. **Hongwei Wang:** Software, Visualization. **Long Cheng:** Funding acquisition, Resources, Supervision, Validation. **Tiantian Xu:** Funding acquisition, Project administration, Resources, Supervision, Validation.

## Declaration of competing interest

The authors declare that they have no conflicts of interest in this work.

## Appendix A Supplementary materials

Supplementary Data S1 Supplementary Raw Research Data. This is open data under the CC BY license http://creativecommons.org/licenses/by/4.0/ Supplementary Data S1

Supplementary Data S2 Supplementary Raw Research Data. This is open data under the CC BY license http://creativecommons.org/licenses/by/4.0/ Supplementary Data S2

Supplementary Data S3 Supplementary Raw Research Data. This is open data under the CC BY license http://creativecommons.org/licenses/by/4.0/ Supplementary Data S3
